# Novel Pt (II) Complexes With Anticancer Activity Against Pancreatic Ductal Adenocarcinoma Cells

**DOI:** 10.1155/bca/5588491

**Published:** 2024-12-31

**Authors:** Erika Stefàno, Gianluca Rovito, Luca G. Cossa, Federica De Castro, Viviana Vergaro, Asjad Ali, Giulia My, Danilo Migoni, Antonella Muscella, Santo Marsigliante, Michele Benedetti, Francesco Paolo Fanizzi

**Affiliations:** ^1^Department of Biological and Environmental Sciences and Technologies (DiSTeBA), University of Salento, Lecce, Via Monteroni I-73100, Italy; ^2^Department of Experimental Medicine, University of Salento, Lecce, Via Monteroni I-73100, Italy

## Abstract

Pancreatic ductal adenocarcinoma (PDAC) is a highly aggressive type of solid tumor that is becoming more common. *cis*-[PtCl_2_ (NH_3_)_2_] (in short cisplatin or CDDP) has been shown to be effective in treating various cancers, including PDAC. However, the development of resistance to chemotherapy drugs has created a need for the synthesis of new anticancer agents. Platinum-based drugs containing the bidentate ligand phenanthroline have been found to have strong antitumor activity due to their ability to cause DNA damage. In this study, we examined the ability of two Pt (II) cationic complexes, [Pt(*η*^1^-C_2_H_4_OR) (DMSO) (phen)]^+^ (in short Pt-EtORSOphen; *R* = Me, **1**; Et, **2**), to inhibit the growth and spread of BxPC-3 PDAC cells, in comparison to CDDP. The length of the alkyl chain and its associated lipophilic properties did not affect the anticancer effects of complexes **1** and **2** in BxPC-3 cells. However, it did appear to influence the rapid loss of mitochondrial membrane potential (ΔΨ_M_), suggesting that these complexes could potentially be used as mitochondria-targeted lipophilic cations in anticancer therapy.

## 1. Introduction

Pancreatic ductal adenocarcinoma (PDAC) is a highly lethal form of cancer. Although it is relatively rare, it ranks among the top causes of death from cancer [1–3]. The mortality rate for PDAC is approximately 90%, largely due to late diagnosis at advanced stages of the disease when symptoms have manifested and the cancer has spread to other areas of the body [4, 5]. Additionally, PDAC is known for its aggressive nature, rapid progression, and resistance to available treatments [6–8].

Chemotherapy is the preferred treatment for patients with advanced pancreatic cancer. Currently, combination chemotherapy regimens are utilized to treat metastatic PDAC, such as combining gemcitabine with other cytotoxic agents, including platinum-based agents [9–13]. However, neither chemotherapy, surgery, nor radiation has shown significant improvements in clinical outcomes [1].

**Figure 1:**
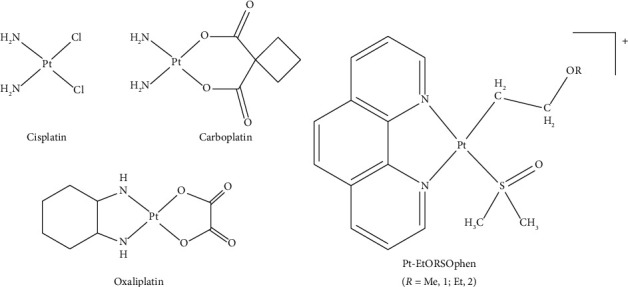
Chemical structure of (a) cisplatin, carboplatin, and oxaliplatin and (b) [Pt (η^1^–C_2_H_4_–OR) (DMSO) (phen)] Cl; R = Me (**1**) and Et (**2**); both compounds are indicated in short as Pt-EtORSOphen.

The need for more effective antitumor drugs has driven the synthesis and study of new platinum compounds over the past fifty years [14–23]. Among these, platinum-based compounds have been extensively studied, but currently only *cis*-[PtCl_2_ (NH_3_)_2_] (in short cisplatin or CDDP), carboplatin, and oxaliplatin have been approved globally [24] (Figure 1). CDDP has a broad spectrum of antitumor activity and is administered to 40%–80% of all cancer patients undergoing chemotherapy for various types of cancer (testicular, ovarian, lung, head and neck, urothelial, and others) [25, 26]. Unfortunately, in patients with PDAC, the administration of CDDP can lead to serious side effects [27, 28]. Since 2011, two combination regimens have become the gold standard for treating metastatic PDAC: 5-fluorouracil (5-FU)/leucovorin with irinotecan and oxaliplatin (FOLFIRINOX), and gemcitabine with nab-paclitaxel. These approaches have shown a slight increase in response rate, progression-free survival rate, and overall survival in patients with PDAC [29, 30]. In 2023, the FDA approved the use of irinotecan liposome in combination with 5-FU/leucovorin and oxaliplatin (NALIRIFOX) as a new first-line treatment for patients with metastatic PDAC [31]. However, the limited efficacy and selectivity of current chemotherapies used in PDAC treatment necessitates the development of new platinum compounds with greater cytotoxicity and selectivity for tumor cells compared to currently used platinum drugs [32–34].

In a recent study, we described the synthesis and cytotoxic activity of a series of platinum complexes, [Pt (*η*^1^-C_2_H_4_OR) (DMSO) (phen)]^+^ (in short Pt-EtORSOphen; *R* = Me, **1**; Et, **2**; Pr; Bu), on various cancer cell lines. The aim was to investigate the impact of alkyl chain length on the antitumoral properties of these complexes. Among the four platinum complexes, **2** showed the most promising results [35]. Interestingly, complex **1** exhibited higher cytotoxicity than CDDP in multiple cancer cell lines, particularly in neuroblastoma cells. Further metabolomic analysis of neuroblastoma cells revealed that the alteration of GSH metabolism [17, 36, 37] is the primary mechanism responsible for the observed cytotoxicity of **1**.

KRAS, TP53, SMAD4, and p16 are dominant mutations in PDAC, with a prevalence of over 50% in patients with this type of cancer [38]. The cell lines MIA PaCa-2, PANC-1, YAPC, and BxPC-3 are commonly used as in vitro models to study the development of PDAC. These cell lines originate from primary tumors and exhibit distinct phenotypic and genotypic characteristics, including mutations in the KRAS, TP53, SMAD4, and p16 genes. These mutations may play a role in the differentiation grade or biological behavior of PDAC cell lines [39, 40].

In three distinct PDAC cell lines (Mia PaCa-2, PANC-1, and YAPC), compounds **1** and **2** exhibited greater antiproliferative effects compared to CDDP. This was particularly evident in YAPC cells, where both compounds significantly induced apoptosis and cell cycle arrest. Additionally, **1** and **2** caused a rapid decrease in mitochondrial membrane potential in YAPC cells, likely due to their cationic and lipophilic nature, in contrast to CDDP [41].

The current study aimed to investigate the effects of [Pt (*η*^1^-C_2_H_4_OR) (DMSO) (phen)]^+^ (in short Pt-EtORSOphen; *R* = Me, **1**; Et, **2**), on BxPC-3 PDAC cell line, in comparison to CDDP. The antiproliferative and antimetastatic properties of **1** and **2** on BxPC-3 were also examined.

The BxPC-3 cell line was chosen for its moderate to poor differentiation grade, similar to YAPC cells, making it a suitable model for studying Pt-EtORSOphen complexes. BxPC-3 cells exhibit unique biological behaviors and mutations typical of many PDAC cases, including KRAS and p53 [42]. This makes them ideal for exploring the molecular mechanisms of PDAC and the response to new treatments. Additionally, BxPC-3 cells grow robustly and consistently replicate in vivo tumor characteristics, ensuring reproducible experimental results. This reliability makes them perfect for evaluating further the antiproliferative and antimetastatic properties of Pt-EtOMeSOphen (**1**) and Pt-EtOEtSOphen (**2**). Including BxPC-3 in our study helps validate and extend findings on these platinum complexes and enhances our understanding of their therapeutic potential for PDAC.

## 2. Materials and Methods

### 2.1. Synthesis of Pt (II) Compounds

Commercially available reagents and solvents were used as received, without further purification. *cis*-[PtCl_2_ (NH_3_)_2_] (in short CDDP) was supplied by Sigma-Aldrich. The two phen-containing complexes, [Pt (*η*^1^-C_2_H_4_OMe) (DMSO) (phen)]^+^ (**1**) and [Pt (*η*^1^-C_2_H_4_OEt) (DMSO) (phen)]^+^ (**2**), were synthesized according to previously published methods, starting from the corresponding [PtCl (*η*^1^-C_2_H_4_OR) (phen)] (*R* = Me, Et) precursors [17, 35]. Specifically, we dissolved 0.05 mmol of Zeise's salt, K[PtCl_3_(*η*^2^-CH_2_=CH_2_)]·H_2_O, in a large excess of alcoholic reagent, ROH, at 0°C using an ice bath. We then added 2.0 mmol of anhydrous Na_2_SO_4_ under magnetic stirring to reduce the amount of dissolved water. Next, we added 0.05 mmol of 1, 10-phenanthroline and 2.0 mmol of Na_2_CO_3_, in stoichiometric amounts. The resulting alcoholic solution was stirred for 24 h and then evaporated under vacuum to yield a yellow solid residue. This was washed multiple times with water to remove any residual salts, filtered, and dried under vacuum to obtain the pure solid products [PtCl (*η*^1^-C_2_H_4_-OMe) (phen)] and [PtCl (*η*^1^-C_2_H_4_-OEt) (phen)]. These solid complexes were then suspended in excess DMSO and stirred at room temperature. The reaction was almost quantitative and after 2 days, a yellow DMSO solution containing the final pure [Pt (*η*^1^-C_2_H_4_-OR) (DMSO) (phen)]Cl {*R* = Me (**1**), Et (**2**)} complex salt was obtained.

The relative stability of Pt-EtOMeSOphen (**1**) complex in the cell culture medium has been evaluated by ^1^H NMR spectra collected, in 90% RPMI 1640 Medium/10% D_2_O, at intervals of 0, 8, 16, 24, 48, and 72 h (see Figure S1).

NMR measurements were performed on a Bruker Avance DPX 400 NMR spectrometer or a Bruker AVANCE III 600 Ascend NMR spectrometer (Bruker, Ettlingen, Germany), equipped with a TCI cryoprobe incorporating a *z*-axis gradient coil and automatic tuning/matching, at a temperature of 300 K. ^1^H NMR monodimensional spectra and [^1^H, ^195^Pt]-HETCOR bidimensional experiments were recorded by using deuterated CDCl_3_ or D_2_O as solvents. The ^1^H NMR spectra were referenced to TMS, and the residual proton signal of the solvent [CDCl_3_; *δ* (^1^H) = 7.24 ppm; D_2_O; *δ* (^1^H) = 4.7 ppm] was used as the internal standard. The ^195^Pt NMR chemical shifts were referenced to H_2_[PtCl_6_] [*δ* (^195^Pt) = 0 ppm] in D_2_O, as the external reference.

### 2.2. Analysis by Inductively Coupled Plasma Atomic Emission Spectroscopy (ICP-AES)

To determine the concentration of platinum, BxPC-3 cells were incubated with 30 μm of Pt (II) compounds for different time points and samples were analyzed by ICP-AES as previously reported [17]. Protein amounts of the treated cells were determined with the Bradford dye-binding method (Bio-Rad protein assay), using lyophilized bovine serum albumin as a standard. The concentration of platinum complexes in PDAC cells was expressed as ng of Pt (II)/mg of protein.

### 2.3. Cell Culture

BxPC-3 cells were cultured as previously reported [41] in a humidified incubator at 37°C with 5% CO_2_ in air.

### 2.4. Cytotoxicity Assay

The viability of PDAC cells was assessed using the SRB (sulforhodamine B) and MTT (3-(4, 5-dimethylthiazol-2-yl)-2,5-diphenyltetrazolium bromide; data not shown) colorimetric assays, as previously reported [41, 43]. Cells were exposed to different concentrations of CDDP, **1**, and **2** for 24 and 48 h. The percentage of surviving cells was determined by calculating the absorbance ratio of the treated cells to the control cells treated with vehicle only. The data presented represent the mean ± standard deviation from eight replicates of three independent experiments.

### 2.5. Cell Cycle Analysis

BxPC-3 cell cycle was studied after treatment with Pt (II) compounds using the nuclear staining dye propidium iodide (PI) (Thermo Fisher Scientific Inc.), as previously described [41].

### 2.6. Annexin V-Fluorescein Isothiocyanate (FITC)/PI Assay

Cell apoptosis induction was assessed using the annexin V-FITC kit (Thermo Fisher Scientific Inc.). BxPC-3 cells were exposed to a concentration of 30 μm (IC_50_ calculated after 24 h of treatment with CDDP) of Pt (II) compounds. The samples were processed as in Stefàno et al. [41] and then analyzed using a flow cytometer (BD Biosciences, San Jose, CA, USA) and BD Accuri C6 Software.

### 2.7. Mitochondrial Membrane Potential (JC-1) Assay

BxPC-3 cells were cultured and incubated with JC-1 fluorescent probe (Enzo Life Sciences, Farmingdale, NY, USA) before being treated with 30 μm Pt (II) compounds [41]. The fluorescence of JC-1 was measured using a Jasco FP-750 Spectrofluorometer (JASCO Corporation, Tokyo, Japan). Fluorescence readings were taken at various time points during each experiment. The ratio of JC-1 fluorescence intensity at 590 and 520 nm was calculated for each time point and used as a qualitative measure of mitochondrial membrane potential (ΔΨ_M_).

### 2.8. Fluorescence Microscopy: Double Staining of Nuclei (DAPI) and Mitochondria (JC-1)

The induction of apoptosis in BxPC-3 cells was assessed using fluorescence microscopy, with JC-1 and DAPI (4, 6-diammine-2-phenylindol) staining. The cells were treated with 30 μm Pt (II) complexes for 24 h and then stained with JC-1 following the protocol described in the previous paragraph. After fixation with 4% (*w*/*v*) paraformaldehyde, the cells were stained with 1 μg/mL DAPI [41]. The EVOS XL Cell Imaging System microscope (Thermo Fisher Scientific, Waltham, MA, USA) was used to analyze cell fluorescence.

### 2.9. Wound Healing Migration Assay

BxPC-3 cells were cultured in 6-well plates (2 × 10^5^ cells/mL) and incubated overnight. The cell monolayer was then disrupted by scraping with a 200 μL micropipette tip, creating a strip approximately 500 μm in diameter. The cells were subsequently washed twice with PBS and complete DMEM medium was added. Sublethal concentrations (0.5–1.5 μm) of CDDP, Pt-EtOMeSOphen (**1**), and Pt-EtOEtSOphen (**2**) were applied to the BxPC-3 cells. The recovery of the monolayer was observed over a period of 24–48 h, and images were captured at different time points using a digital camera. The migrated area was measured using Photoshop CS6 software. At least six fields per dish were analyzed, and the width of the injury line was reported as a percentage of that in untreated control cells.

### 2.10. 3D Tumor Spheroid-Based Migration Assay

Tumor spheroid-based migration assays were conducted to assess the effectiveness of Pt complexes [44]. U-shaped 96-well plates were seeded with 5000 BxPC-3 cells per well and centrifuged to promote cell aggregation. Plates were incubated at 37°C for 72 h to allow for spheroid formation. Sublethal doses of CDDP, **1**, and **2** complexes were applied to the spheroids, and the migration area was determined using optical analysis with Adobe Photoshop CS6 software [45]. After 6 days, cell viability was measured using MTT assay. The spheroids were incubated with a 5 mg/mL MTT solution for 1 day, followed by the addition of 100 μL of isopropanol to each well. The plates were then incubated for 4 h to dissolve the formazan crystals. Absorbance at 550 nm was measured to determine cell viability.

### 2.11. Gelatin Zymography

The impact of CDDP, Pt-EtOMeSOphen (**1**), and Pt-EtOEtSOphen (**2**) on the activity of metalloproteinases (MMPs) was investigated using gelatin zymography. BxPC-3 cells were cultured in 35 mm Petri dishes (4 × 10^5^ cells per dish) and treated with sublethal concentrations (0.5–1.5 μm) of Pt (II) compounds for 48 h. The supernatants were then collected and centrifuged at 10,000 g for 10 min at 4°C to remove cellular debris. Each supernatant sample (35 μL) was diluted with a 4X SDS sample buffer and analyzed by electrophoresis on 10% (*wt*/*v*) polyacrylamide gels containing 1 mg/mL gelatin as a substrate for metalloproteases. After electrophoreses, the gels were washed with renaturation buffer for 1 h and with developing buffer at 37°C overnight. The gels were then stained and destained [37], and the gelatinolytic activity was observed as horizontal white bands on a blue background. The intensity of the bands was measured using Image Lab software (Bio-Rad).

### 2.12. Statistical Analyses

Statistical analyses were conducted using GraphPad Prism 8 software (GraphPad Software, San Diego, CA, USA). The normality of the data was confirmed prior to analysis using the Kolmogorov–Smirnov tests. Statistical analysis was performed using ANOVA with Tukey's multiple comparisons test.

## 3. Results

### 3.1. Cytotoxicity of cis-[PtCl_2_(NH_3_)_2_] and [Pt(η^1^-C_2_H_4_OR)(DMSO)(phen)]^+^ (R = Me, 1; Et, 2) on PDAC cells

**Figure 2:**
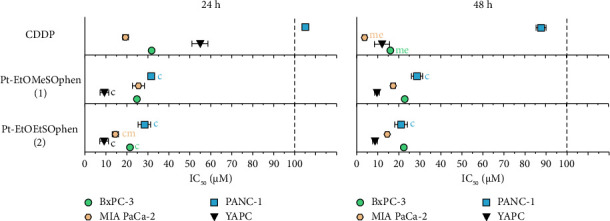
Cytotoxic effects of Pt (II) compounds on four PDAC cell lines. *cis*-[PtCl_2_ (NH_3_)_2_] (in short CDDP) and [Pt (*η*^1^−C_2_H_4_OR) (DMSO) (phen)]^+^ (in short Pt-EtORSOphen; *R* = Me, **1**; Et, **2**) complexes were tested at concentrations of 1–100 μm on four PDAC cell lines (BxPC-3, MIA PaCa-2, PANC-1, and YAPC cells) for 24 and 48 h. Cell viability was determined using the SRB assay and IC_50_ values were calculated and presented in the graphs. IC_50_ values above the dotted line were not determined as they exceeded the maximum tested dose (> 100 μm). Data represent the mean ± standard deviation of three experiments, each with eight replicates, and are presented as a percentage of control. Letters indicate IC_50_ values that are significantly lower (*p* < 0.05) than those of CDDP (c), Pt-EtOMeSOphen (**1**) (m), and Pt-EtOEtSOphen (**2**) (e) at the same time point.

The cytotoxic activity of the two Pt-EtORSOphen (*R* = Me, **1**; Et, **2**) compounds was assessed using the SRB assay on four human PDAC cell lines (BxPC-3, Mia PaCa-2, PANC-**1**, and YAPC) and compared to that of *cis*-[PtCl_2_ (NH_3_)_2_] (in short CDDP). Both CDDP and the phen-containing complexes exhibited a significant inhibitory effect on PDAC cell viability in a time- and dose-dependent manner (Figure 2). In general, **1** and **2** demonstrated a higher and faster cytotoxic effect compared to CDDP in the tested cell lines, as previously observed in other types of cancer cells [17, 35]. After 48 h of incubation, CDDP showed greater cytotoxicity in BxPC-3 (IC_50_ = 16.1 ± 1.0 μm) and MIA PaCa-2 (IC_50_ = 3.8 ± 1.1 μm) cells [41], while the phen-containing complexes had comparable effects (Figure 2). However, CDDP had similar effects on YAPC cells. PANC-1 cells appeared to be the most resistant to CDDP among the tested cell lines (IC_50_ after 24 h > 100; IC_50_ after 48 h = 87.9 ± 2.3 μm) [41]. Interestingly, **1** and **2** were highly effective (*p* < 0.05) against this cell line (Figure 2).

To assess the cytotoxicity of Pt (II) complexes toward cancer cells compared to normal cells, a selectivity index (SI) was calculated for the four tested cancer cell lines. Both CDDP and phen-containing complexes showed selectivity against PDAC cancer cells compared to normal HK-2 cells, except for PANC-1 cells where CDDP was not selective (SI < 1) and complex **1** showed equal cytotoxicity (SI = 1 after 48 h). Complexes **1** and **2** appeared to be less selective than CDDP in BxPC-3 and MIA PaCa-2 cells, but more selective in PANC-1 and YAPC cell lines (see Figures S21 and S3).

**Figure 3:**
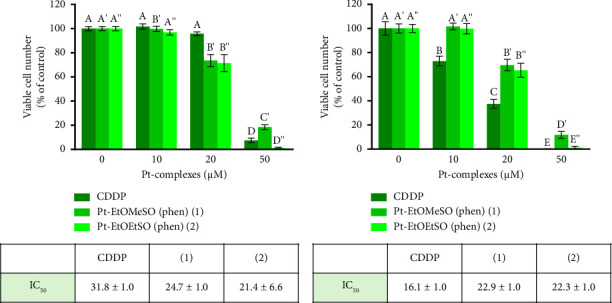
Cytotoxic effects of *cis*-[PtCl_2_ (NH_3_)_2_] (in short CDDP) and [Pt (*η*^1^-C_2_H_4_OR) (DMSO) (phen)]^+^ (in short Pt-EtORSOphen; *R* = Me, **1**; Et, **2**) on BxPC-3 cells. BxPC-3 cells were exposed to varying concentrations (1–50 μm) of CDDP, **1**, and **2** for (a) 24 and (b) 48 h. The IC_50_ values were calculated and are presented in the tables as means ± standard deviation from eight replicate wells per microliter plate, repeated three times. Values with the same letter are not significantly different according to Tukey's multiple comparisons test.

Based on the phenotypic and genotypic characteristics of four cell lines, we have chosen to further investigate the antitumoral effects of Pt-EtORSOphen (**1** and **2**) complexes on BxPC-3 cells. This decision was based on the fact that BxPC-3 cells have a similar differentiation grade (moderate to poor) to YAPC cells [27, 40, 46], which have previously shown high cytotoxic activity when treated with **1** and **2** [41]. Additionally, the comparable cytotoxic effects of the two Pt-EtORSOphen complexes in BxPC-3 and YAPC cells, as compared to MIA PaCa-2 and PANC-1 cells, suggest a potential shared mechanism of action in these two pancreatic cell lines (see Figure 2) [41]. In fact, the induction of cell death by **1** and **2** was not significantly different in YAPC and BxPC-3 cells (with an IC_50_ value of approximately 9 μm in YAPC and 22 μm in BxPC-3 for both compounds) (*p*  >  0.05) (Figure 3) [41].

Exposure of BxPC-3 cells to platinum compounds at concentrations ranging from 1 to 50 μm caused a concentration-dependent reduction in cell viability (see Figure 3). After 24 h of treatment, **1** and **2** caused higher levels of cell death compared to CDDP. After 48 h of incubation, the cytotoxicity increases for CDDP, but remains almost unvaried for complexes **1** and **2** (see Figure 3). Additionally, the percentage of cell viability was slightly lower after incubation with the complex containing an ethyl group (**2**) compared to the complex with a methyl group (**1**).

### 3.2. Intracellular Accumulation of Pt (II) Complexes

**Figure 4:**
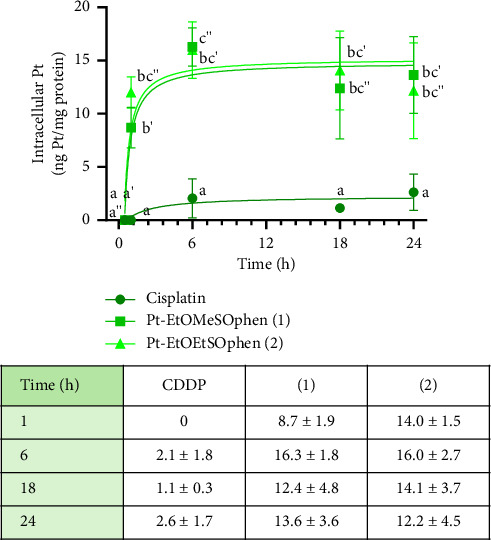
Intracellular uptake of [Pt (*η*^1^-C_2_H_4_OR) (DMSO) (phen)]^+^ (in short Pt-EtORSOphen; *R* = Me, **1**; Et, **2**) and *cis*-[PtCl_2_ (NH_3_)_2_] (in short CDDP) in BxPC-3 cells. Cells were exposed to 30 μm of each Pt (II) compound for 1, 6, 18, and 24 h. Total intracellular accumulation was measured by inductively coupled plasma atomic emission spectroscopy (ICP-AES). Each data point represents the mean ± SD of three independent experiments and is expressed as ng of Pt (II)/mg of protein. Letters indicate significant differences (*p* < 0.05).

The total amount of platinum inside BxPC-3 cells was measured using ICP-AES after incubation with 30 μm of CDDP, **1**, and **2** (with an IC_50_ value calculated after treatment with CDDP) for 1–24 h. The platinum concentration inside cells was expressed as ng of Pt (II)/mg of protein. The intracellular levels of complexes **1** and **2** were found to be higher than CDDP after just 1 h of incubation (as shown in Figure 4), consistent with previous observations in YAPC cells [41]. After 24 h of treatment, the accumulation of complexes **1** and **2** was approximately five times higher than CDDP (*p* < 0.05) (Figure 4). However, this high intracellular content of platinum in BxPC-3 cells, for both **1** and **2**, did not correspond to increased cytotoxicity (as shown in Figure 3), as their antiproliferative activity was not higher than that of CDDP, which enters cells to a lesser extent (Figure 4). Nevertheless, the rapid induction of cytotoxicity by complexes **1** and **2** compared to CDDP (Figure 3) may be related to their high intracellular accumulation.

The comparable IC_50_ value (*p* > 0.05) determined at 24–48 h (Figure 3) and the overlapping platinum accumulation profile enabled us to conduct subsequent analyses using identical treatment doses for both **1** and **2**. The results obtained from treatment with the two phen-containing complexes did not show significant differences (Figures S4–S6). Therefore, only the results obtained from one of these complexes, **2**, are presented here.

### 3.3. Pt-EtOMeSOphen (**1**) and Pt-EtOEtSOphen (**2**) Induce Apoptosis and Cause Cell Cycle Arrest in BxPC-3 Cells

**Figure 5:**
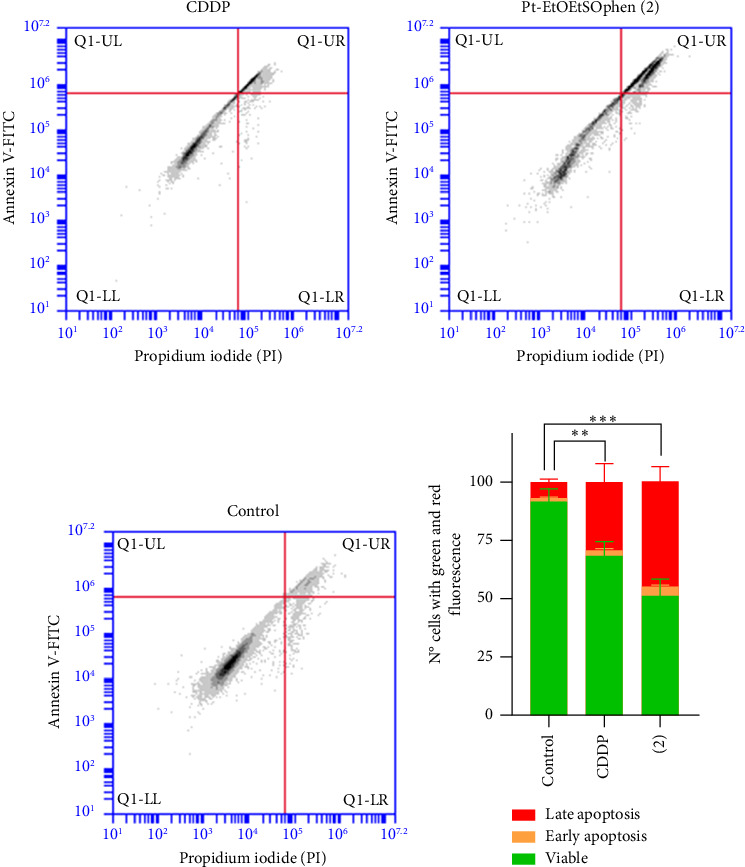
[Pt (*η*^1^-C_2_H_4_OEt) (DMSO) (phen)]^+^ (in short Pt-EtOEtSOphen; **2**) and *cis*-[PtCl_2_ (NH_3_)_2_] (in short CDDP) induced apoptosis in BxPC-3 cells. (a–c) Cell death was quantified by flow cytometry after annexin V-FITC/PI staining. BxPC-3 cells were treated with or without 30 μM (a) CDDP and (b) complex **2** for 24 h. Q1-UL, PI+ (necrotic cells); Q1-UR, annexin V-FITC + PI+ (cells in late apoptosis and necrosis); Q1-LR, annexin V-FITC + PI− (cells in early apoptosis); Q1-LL, annexin V-FITC − PI− (living cells). The percentage of viable and dead cells was quantified using BD Accuri C6 software and displayed as a bar graph. (d) Asterisks (⁣^∗∗^*p* < 0.01; ⁣^∗∗∗^*p* < 0.001) indicate values of viable cells that are significantly different from untreated cells after exposure to CDDP and complex **2**.

**Figure 6:**
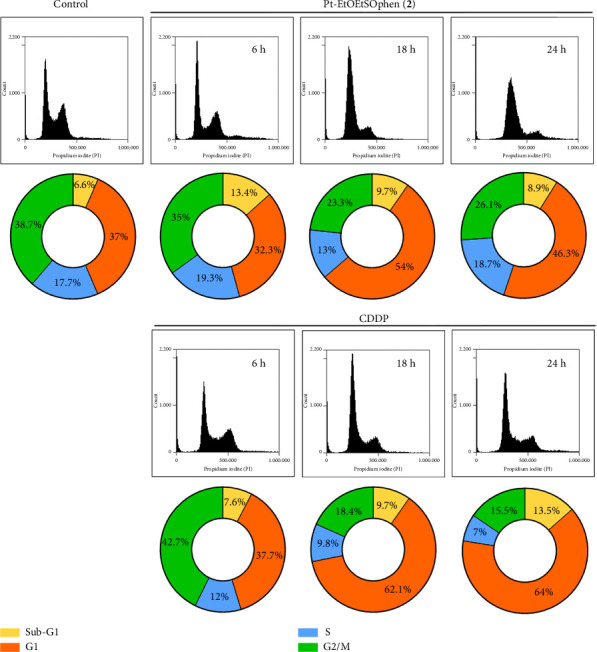
Effects of *cis*-[PtCl_2_ (NH_3_)_2_] (in short CDDP) and [Pt (*η*^1^-C_2_H_4_OEt) (DMSO) (phen)]^+^ (in short Pt-EtOEtSOphen; **2**) on the cell cycle of BxPC-3 cells. Cell cycle distribution was analyzed using flow cytometry (BD Accuri C6 flow cytometer) in PI-stained cells after 6, 18, and 24 h of treatment with or without CDDP and Pt-EtOEtSOphen (**2**). The pie charts indicate the percentages of cells in the G1, S, or G2/M phases of the cell cycle.

**Figure 7:**
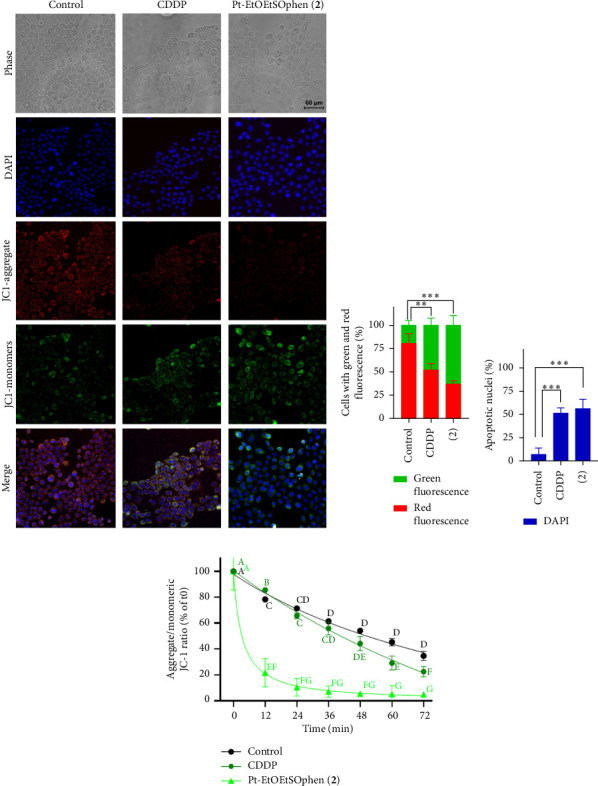
Analysis of Pt (II) complex–induced apoptosis using fluorescence microscopy and fluorescence spectroscopy. (a–d) Double staining of nuclei (DAPI) and mitochondria (JC-1) was performed on BxPC-3 cells using fluorescence microscopy. Cells were treated with or without 30 μm *cis*-[PtCl_2_ (NH_3_)_2_] (in short CDDP) and complex [Pt (*η*^1^-C_2_H_4_OEt) (DMSO) (phen)]^+^ (in short Pt-EtOEtSOphen; **2**) for 24 h and then stained with DAPI and JC-1 dyes. Quantification of (a, b) green/red fluorescence for measuring mitochondrial membrane potential (ΔΨ_M_) and (a, c) blue fluorescence for apoptotic nuclei was carried out using ImageJ software. Asterisks indicate values that are significantly different (⁣^∗∗^*p* < 0.01; ⁣^∗∗∗^*p* < 0.001). (d) Measurements of *J*-aggregate and *J*-monomer fluorescence were also obtained by fluorimetry. Data are expressed as the change in 590/520 nm fluorescence ratio induced by treatment relative to the initial (control) 590/520 nm ratio. Results are presented as the mean ± SD of three independent experiments. Values with shared letters are not significantly different according to Tukey's multiple comparisons test.

The effects of CDDP, **1**, and **2** on apoptosis and cell cycle arrest were examined using flow cytometry with annexin V and PI assays (Figures 5 and 6). Additionally, apoptotic cells were visualized through DAPI staining under fluorescent microscopy (Figure 7). The role of mitochondria in BxPC-3 cell death was also investigated by fluorescent JC-1 staining, as mitochondrial membrane potential is an indicator of mitochondrial function and cell viability (Figure 7 and Figures S4–S6).

Annexin V-FITC/PI staining was utilized to quantify apoptosis in PDAC cells (Figure 5 and Figure S4). BxPC-3 cells were treated with 30 μm of CDDP, **1**, and **2** complexes for 24 h to assess their ability to induce cell death compared to untreated cells (Figure 5 and Figure S4). Treatment with CDDP and **2** resulted in a significant increase in apoptosis (32% and 49%, respectively) compared to the control (*p* < 0.01). Additionally, complex **2** induced cell death more effectively than CDDP (*p* < 0.05) after 24 h, confirming the rapid antiproliferative activity of these novel compounds (Figure 5) [17, 35, 37].

The effect of Pt (II) complexes on the cell cycle of BxPC-3 cells was assessed using flow cytometry with PI staining after 6, 18, and 24 h of treatment (Figure 6 and Figure S5). The results were compared to those obtained after incubation with CDDP, which served as a positive control. Treatment with **2** did not result in a significant increase in the percentage of PDAC cells in the sub-G1 (indicative of cell death) compared to CDDP. However, compound **2** did decrease the percentage of cells in the G2/M phase, although to a lesser extent than CDDP. Treatment with CDDP led to a two-fold increase in the percentage of cells in the G1 phase compared to complex **2**, likely due to the extensive DNA damage induced by CDDP [47].

During apoptosis, the opening of mitochondrial permeability pores and loss of the electrochemical gradient are associated with a reduction in mitochondrial membrane potential (ΔΨ_M_). Therefore, ΔΨ_M_ serves as a crucial indicator of mitochondrial function and can be used as a parameter for assessing cell health [48]. The measurement of ΔΨ_M_ was performed using fluorescence microscopy and fluorometric analysis, utilizing the JC-1 fluorescence probe (Figure 7 and Figure S6). In healthy cells, JC-1 forms *J*-aggregate complexes (red fluorescence, high ΔΨ_M_), while in apoptotic cells, the depolarization of the mitochondrial membrane potential provokes the dissociation of the aggregate into the monomeric form of the JC-1 dye, causing a shift in fluorescence to 530 nm (green fluorescence, low ΔΨ_M_). Thus, the ratio of red to green fluorescence intensity correlates with changes in ΔΨ_M_ and can be used as an indicator of the induction of the apoptotic process [49].

The dual staining technique using JC-1 and DAPI revealed an increase in apoptotic cells after 24 h of incubation with CDDP and **2**, compared to control cells (*p* < 0.01) (Figures 7(a) and 7(b)). This is consistent with the results from flow cytometry (Figure 6), which showed a higher percentage of apoptotic BxPC-3 cells after treatment with **2** (63%), compared to CDDP (48%) (*p* < 0.05). Furthermore, the percentage of apoptotic nuclei was significantly higher in cells treated with platinum agents compared to controls (*p* < 0.05), although there was no significant difference between CDDP- and **2**-treated cells (Figure 7(b)). Fluorometric analysis confirmed an early decrease in mitochondrial membrane potential (ΔΨ_M_) as indicated by a shift toward green fluorescence, which was observed as early as 12 min after treatment with **2** (Figure 7(d)). In contrast, CDDP caused a slower decrease in ΔΨ_M_.

### 3.4. Pt (II) Complex Effects on Cell Migration

**Figure 8:**
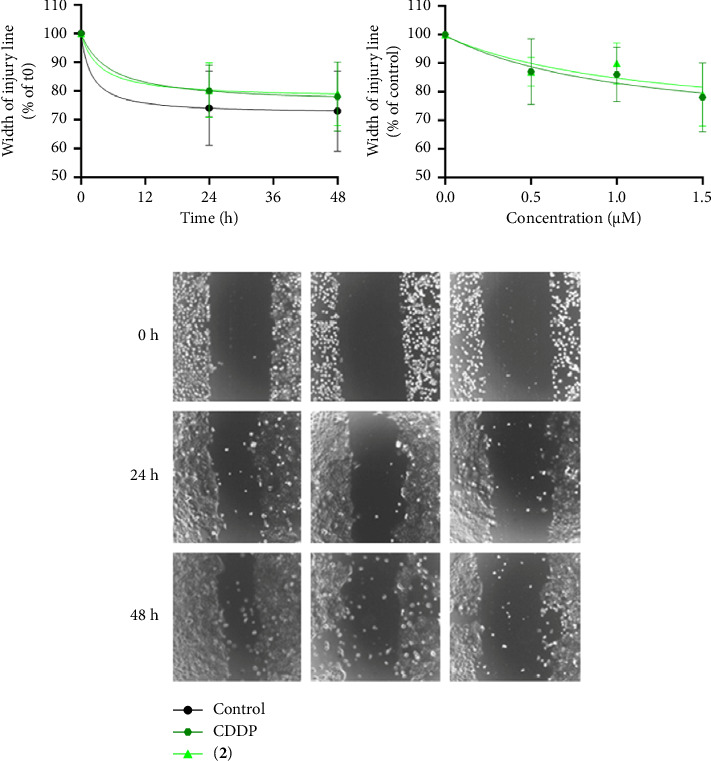
Antimigratory ability of Pt (II) complexes on BxPC-3 cells. (a–c) The migratory capacity of BxPC-3 cells was assessed using the bidimensional wound healing assay after exposure to sublethal doses of *cis*-[PtCl_2_ (NH_3_)_2_] (in short CDDP) and [Pt (*η*^1^-C_2_H_4_OEt) (DMSO) (phen)]^+^ (in short Pt-EtOEtSOphen; **2**). Cells were treated with different concentrations (0.5, 1, and 1.5 μm) of Pt (II) compounds for 48 h and monitored by microscopy. (a) The percentage of wound closure (means ± SD) was quantified at 24 and 48 h and normalized to 100% wound width at t0 (*n* = 4). (b) The effect of different concentrations of Pt (II) complexes on BxPC-3 cell migration was analyzed by measuring wound width at 48 h and normalizing it to control cells. (c) Bright field images were collected using a 10x objective and representative images of wounds at 0, 24, and 48 h were included.

The migratory ability of BxPC-3 cells was assessed following exposure to sublethal concentrations (0.5–1.5 μm) of CDDP, Pt-EtOMeSOphen (**1**) (data not shown), and Pt-EtOEtSOphen (**2**) (Figure 8) using both 2D (wound healing assay) and 3D (tumor spheroid-based assay) migration assays.

In 2D wound healing–based tumor migration assay, both CDDP and **2** (1.5 μm) were found to mildly hinder cell migration in dose-dependent manner over a 48 h incubation period (Figure 8(b)), although this effect was not statistically significant compared to untreated cells (*p* > 0.05) (Figures 8(a) and 8(c)).

**Figure 9:**
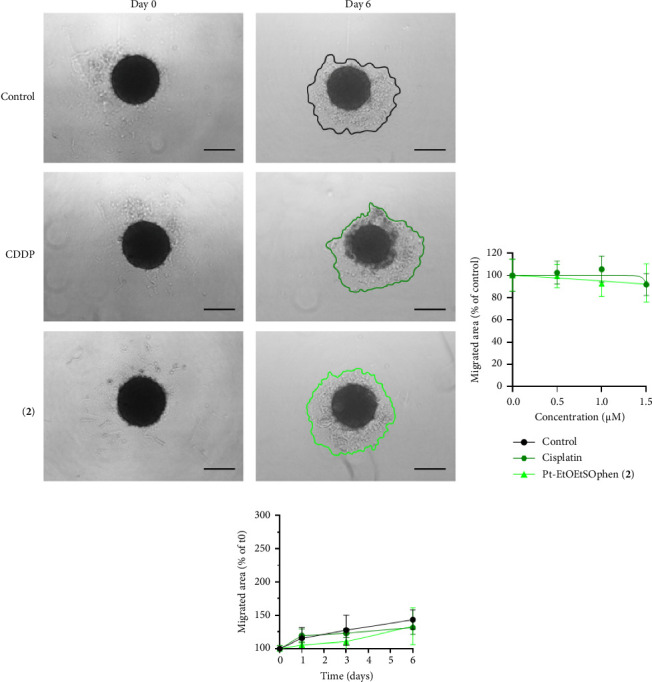
Effects of Pt (II) complexes on 3D tumor spheroids. (a) Digital images of the spheroids captured at 0 and 6 days after exposure to 1.5 μm *cis*-[PtCl_2_ (NH_3_)_2_] (in short CDDP) and [Pt (*η*^1^-C_2_H_4_OEt) (DMSO) (phen)]^+^ (in short Pt-EtOEtSOphen; **2**) (scale bar = 300 μm). (b) Tumor spheroids were treated or not with 0.5–1.5 μm Pt (II) complexes for 6 days and migrating areas were measured and reported on the graph as a percentage of the control. (c) Tumor spheroids were treated or not with 1.5 μm CDDP and 2 at 1, 3, and 6 days and migrating areas were measured and reported on the graph as a percentage of measurement at day 0. All data were expressed as mean ± standard deviation (SD) values of four experiments.

Consistently, the 3D tumor spheroid-based migration assay did not exhibit a significant decrease in migration area following incubation with 0.5–1.5 μm of CDDP and complex **2** (*p* > 0.05) (Figure 9). The viability of tumor spheroids after 6 days of exposure to Pt (II) compounds was not significantly affected (*p* > 0.05; data not shown).

**Figure 10:**
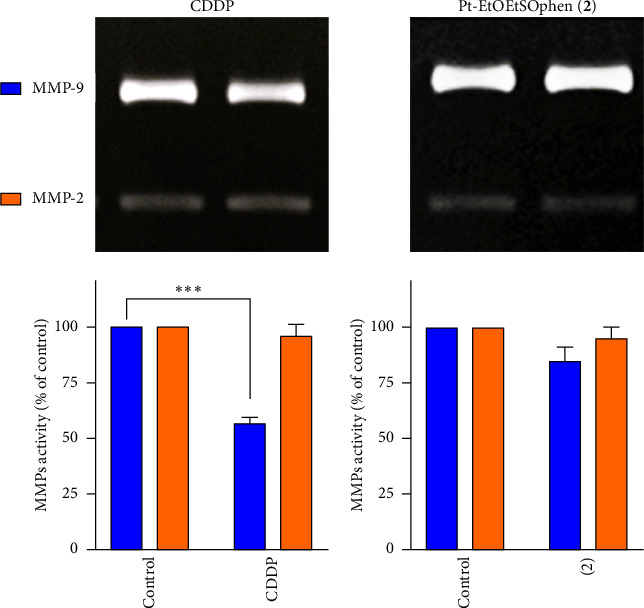
Effects of *cis*-[PtCl_2_ (NH_3_)_2_] (in short CDDP) and [Pt (*η*^1^-C_2_H_4_OEt) (DMSO) (phen)]^+^ (in short Pt-EtOEtSOphen; **2**) on metalloproteinase activity. Cells were treated with or without 1.5 μm of (a) CDDP and (b) Pt-EtOEtSOphen (**2**) for 48 h. Conditioned media were analyzed using gelatin zymography to measure the gelatinolytic activities of metalloproteinases MMP-2 and MMP-9. Results from densitometry are presented as mean ± SD of the sum of gray level values (⁣^∗∗∗^*p* < 0.001 by one-way ANOVA; *n* = 2).

Together with 2D wound healing and 3D tumor spheroid–based migration assays, the measurement of metalloproteinase (MMP-2 and MMP-9) activities was evaluated to better understand the antimetastatic role of CDDP, **1**, and **2** (Figure 10). Exposure of BxPC-3 cells to sublethal dose (1.5 μm) of CDDP resulted in a significant decrease in MMP-9 protein levels, while there were no significant changes in MMP-2 activity. The reduction in MMP-2 activity was minimal compared to the control (*p* > 0.05). In contrast, treatment with 1.5 μm of **2** did not result in a significant reduction in MMP activity (Figure 10).

## 4. Discussion and Conclusions

PDAC is the most prevalent form of pancreatic cancer, accounting for approximately 90% of cases [4, 50]. Its development involves multiple stages, resulting from alterations in various oncogenes and tumor suppressor genes, such as KRAS, TP53, CDKN2A, EGFR, and SMAD4. These genetic changes initially lead to acinar-to-ductal metaplasia, followed by low- and high-grade pancreatic intraepithelial neoplasia, dysplasia, in situ carcinoma, and ultimately invasive carcinoma, which can become resistant to chemotherapeutic agents [51, 52]. The high mortality rate of this type of tumor is partly due to delayed diagnosis and the aggressive behavior of malignant cells, which can spread to nearby tissues early in the disease, making treatment challenging [53, 54]. CDDP has shown significant effectiveness in treating various cancers, including PDAC. However, its use is limited by both inherent and acquired drug resistance in PDAC [11].

We previously investigated the cytotoxic effects of CDDP and two Pt (II) complexes of the type [Pt (*η*^1^-C_2_H_4_OR) (DMSO) (phen)]^+^ (in short Pt-EtORSOphen; *R* = Me, **1**; Et, **2**), on three different PDAC cell lines (Mia PaCa-2, PANC-1, and YAPC), which have distinct phenotypic features such as adhesion, migration, invasion, and tumorigenesis, as well as genotypic differences [40]. Specifically, we focused on the effects of **1** and **2** on YAPC cells, which showed a better response to treatment with phen-containing complexes compared to CDDP [41]. In this study, we expanded our investigation to a fourth PDAC cell line, BxPC-3, which has a similar differentiation grade (moderate to poor) as YAPC cells [27, 40, 46]. We also calculated the SI of CDDP and phen-containing complexes (**1** and **2**) comparing the IC_50_ values for PDAC and normal HK-2 cells. Our results demonstrate a high selectivity of **1** and **2** against cancer cells, as compared to healthy cells (Figure S2 and Figure S3). We found that complex **2** was more cytotoxic than CDDP in all tested cell lines after 24 h of exposure, while complex **1** was more effective in two of the four cell lines (PANC-1 and YAPC). Interestingly, after 48 h of treatment, the two complexes remained more cytotoxic only in PANC-1, on which they also demonstrated to be more selective compared to CDDP (Figure 2 and Figure S3), which is known to have a higher migration/invasion and angiogenic potential compared to the other PDAC cell lines [40, 55]. Previous research by Kowalski and colleagues has shown that organic ligands such as quinolone and phenanthroline in vanadium complexes can selectively induce both apoptosis and necroptosis in PANC-1 cells. Overall, the two complexes showed similar cytotoxic activity, except for MIA PaCa-2, where complex **2** (*R* = ethyl) exhibited higher cytotoxicity than its analog **1** (*R* = methyl) (IC_50_ = 15 μm for **2** and 26 μm for **1**; *p* < 0.05) (Figure 2) [41]. Our recent findings have also shown that in the series of Pt-EtORSOphen (*R* = Me, Et, Pr, Bu) complexes, the presence of an Et moiety (**2**) resulted in higher cytotoxicity in hepatocarcinoma cells (Hep-G2) compared to other tested cell lines. This indicates that the variation in the length of the *η*^1^-C_2_H_4_-OR chain in these novel Pt (II) cationic complexes can impact their ability to inhibit cell growth, resulting in notable variations in cytotoxicity based on the specific type of cancer cells [35, 56]. In both YAPC and BxPC-3 cells, CDDP exhibited a similar cytotoxic effect after 48 h of incubation (IC_50_ = 12 and 16 μm, respectively; *p* > 0.05). However, compounds **1** and **2** showed cytotoxic activity comparable to CDDP in YAPC cells, but were less effective in BxPC-3 cells (Figures 2, 3, and 5) [41]. Additionally, treatment with Pt-EtORSOphen compounds resulted in higher cytotoxicity and sub-G1 phase arrest in YAPC cells compared to BxPC-3 cells (Figure 6) [41]. Interestingly, the induction of cell death in BxPC-3 cells did not appear to be related to the intracellular accumulation of Pt-EtORSOphen complexes, as their content was approximately five times lower than that of CDDP after 24 h (Figure 4), despite similar levels of cytotoxicity (Figures 2 and 3). However, the rapid uptake of Pt-EtORSOphen complexes, producing a higher increase of intracellular Pt (II) concentration, may be correlated with their faster reduction of cell viability compared to CDDP (Figures 2, 3, 4, and 5).

The similarities between YAPC and BxPC-3 suggest that they may be vulnerable to exposure to CDDP. However, differences in the alteration of molecular pathways may affect the behavior of these new Pt (II) compounds, which appear to act through a distinct mechanism that probably is not primarily focused on damaging DNA. Sun and colleagues discovered that structurally similar phen-containing complexes, such as [Pt (OCOCH_2_OR)_2_(phen)], can halt the progression of the human colorectal carcinoma cell cycle in the S or G2/M phases and induce cell death through the apoptosis pathway. These complexes interact with DNA in a slightly different manner than CDDP [56]. We hypothesize that the rapid induction of cell death after exposure to compounds **1** and **2** may be due to their interaction with potential cytosolic targets [17, 36, 37]. Additionally, we observed that **1** and **2** cause damage to mitochondria in both YAPC [41] and BxPC-3 cells (Figure 7). This results in a rapid decrease in mitochondrial membrane potential (ΔΨ_M_) within the first few minutes of incubation, compared to CDDP. Metal-based compounds can directly affect mitochondria, leading to a loss of membrane potential and the release of apoptotic proteins [57–59]. Furthermore, it has been reported that the mitochondrial membrane potential (ΔΨ_M_) in cancer cells is higher than in normal cells, making them more susceptible to the accumulation of cationic lipophilic compounds that target negatively charged mitochondria [60, 61].

In accordance with our previous findings on YAPC cells [41], we observed a significant increase in G1 phase accumulation of BxPC-3 cells following exposure to CDDP, compared to phen-containing complexes (Figure 6). This can be attributed to the DNA damage caused by CDDP. The induction of DNA damage by CDDP can activate proteins such as ATM and ATR, which are involved in cell cycle checkpoint regulation in response to DNA double-strand breaks. This leads to cell cycle arrest at both the G1 and G2/M checkpoints, allowing for DNA repair and cell survival [47]. The activation of ATM and ATR proteins has been linked to CDDP resistance in PDAC following DNA damaging treatment [62]. On the other hand, numerous studies have shown that Pt (II) complexes containing 1, 10-phenanthroline exert their antitumor effects by inserting their aromatic rings into the minor groove of DNA in A-T rich regions [63], causing distortion of the DNA through covalent binding [56]. Additionally, Sun et al. observed that the alkyl chain of [Pt (OCOCH_2_OR)_2_ (phen)] compounds (*R* = Me, Et, Pr, Bu) influences their in vitro antitumor activity. Specifically, complexes with a smaller alkoxy moiety in the leaving group were found to be more active than those with a longer one [56]. Our recent research has shown that complex **2**, with an ethyl group, exhibited greater cytotoxicity in a series of [Pt (*η*^1^-C_2_H_4_-OR) (DMSO) (phen)]Cl (*R* = Me, **1**; Et, **2**; Pr, Bu) complexes in various cell lines. This suggests potential for structural optimization and development of new anticancer drugs [35]. However, we did not observe specific enhanced antitumor activity of complexes with ethyl (**2**) with respect to methyl (**1**) moiety on BxPC-3 cells.

Finally, while sublethal concentrations of **1** and **2** demonstrated antimetastatic activity on neuroblastoma (SH-SY5Y) and PDAC (YAPC) cells [37, 41], they were unable to inhibit migration in BxPC-3 cells (Figures 8 and 9), likely due to differences in their adhesion and migratory/invasive properties [40]. As decreased levels of MMPs are considered an important indicator of a compound's antitumorigenic potential, we also evaluated the activity of gelatinases (matrix metalloproteinase-2 and -9) in BxPC-3 cell-conditioned media. The Pt-EtORSOphen compounds did not reduce gelatinase activity, while CDDP significantly reduced MMP-9 gelatinolytic activity (Figure 10). MMP-2 and MMP-9 are known for their ability to degrade extracellular matrix components, and their altered expression and/or activity in the tumor microenvironment has been linked to PDAC progression [64]. In BxPC-3 cells, the antimetastatic effects were correlated with a reduction in MMP-9 levels [65].

In conclusion, the Pt-EtOMeSOphen (**1**) and Pt-EtOEtSOphen (**2**) complexes exhibited distinct antiproliferative and antimetastatic effects on PDAC cells. In BxPC-3 cells, the length of the alkyl chain and its associated hydrophobic properties did not affect the anticancer effects, but may facilitate uptake of these Pt (II) compounds by cancer cell mitochondria, making them potential candidates for mitochondria-targeted lipophilic cations in anticancer therapy. Traditional chemotherapies, including platinum-based approaches that target DNA replication in rapidly dividing cells, have had limited success in treating tumors due to their lack of specificity for tumor cells. Therefore, investigating new anticancer agents that target different cancer cell pathways may offer a promising approach for selective tumor cell elimination.

## Data Availability

All data supporting the results are included in the article and in the Supporting Information.

## Supporting Information

Figure S1: ^1^H NMR spectra of Pt-EtOMeSOphen (**1**) complex in 90% RPMI 1640 Medium/10% D_2_O; recorded at intervals of 0; 8; 16; 24; 48; and 72 h. Figure S2: cytotoxic effects of *cis*-[PtCl_2_(NH_3_)_2_] (in short CDDP); [Pt (*η*^1^-C_2_H_4_OR) (DMSO) (phen)]^+^ (in short Pt-EtORSOphen; *R* = Me; **1**; Et; **2**); on normal cells. Figure S3: the selectivity index (SI) values of CDDP; Pt-EtOMeSOphen (**1**); and Pt-EtOEtSOphen (**2**). Figure S4: supporting information flow cytometry data showing the induction of apoptosis in BxPC-3 cells by Pt-EtOMeSO(phen) (**1**) and Pt-EtOEtSO(phen) (**2**). Figure S5: supporting information cell cycle distribution data obtained by flow cytometry analysis; showing the effects of Pt-EtOMeSOphen (**1**) and Pt-EtOEtSOphen (**2**) on cell cycle progression. Figure S6: supporting information analysis of Pt (II) complex–induced apoptosis obtained by fluorescence microscopy.
