# Supplementary material for: Novel Pt (II) Complexes With Anticancer Activity Against Pancreatic Ductal Adenocarcinoma Cells
Source: Bioinorg Chem Appl. 2024 Dec 31;2024:5588491. doi: 10.1155/bca/5588491 (PMC11779987; doi:10.1155/bca/5588491)
Supplement: Supporting Information — Additional supporting information can be found online in the Supporting Information section. [file 5588491.f1.docx]

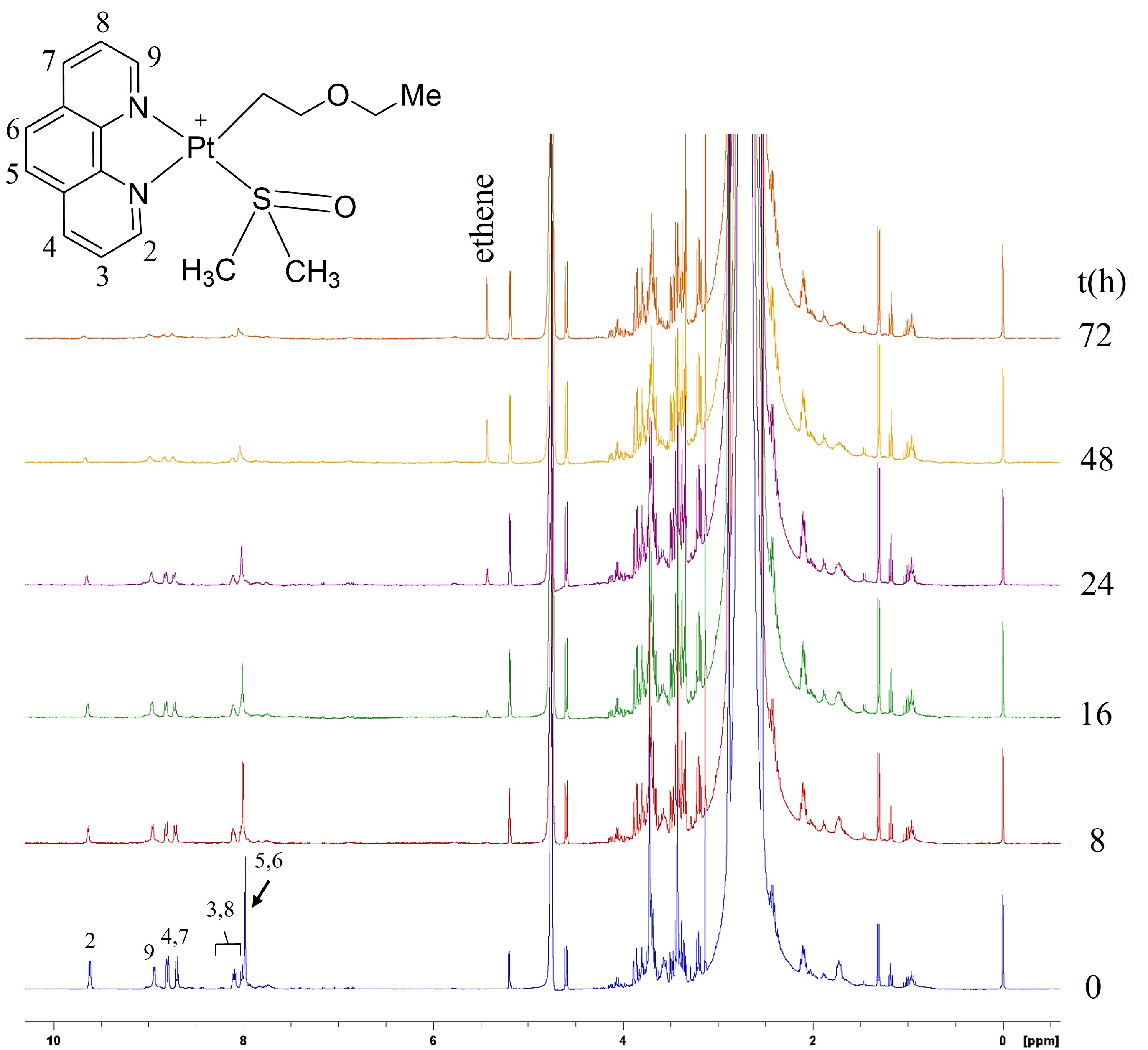


**Figure S1.** ^1^H NMR spectra of Pt-EtOMeSOphen (**1**) complex in 90% RPMI 1640 Medium/10% D_2_O recorded at intervals of 0, 8, 16, 24, 48, and 72 h.


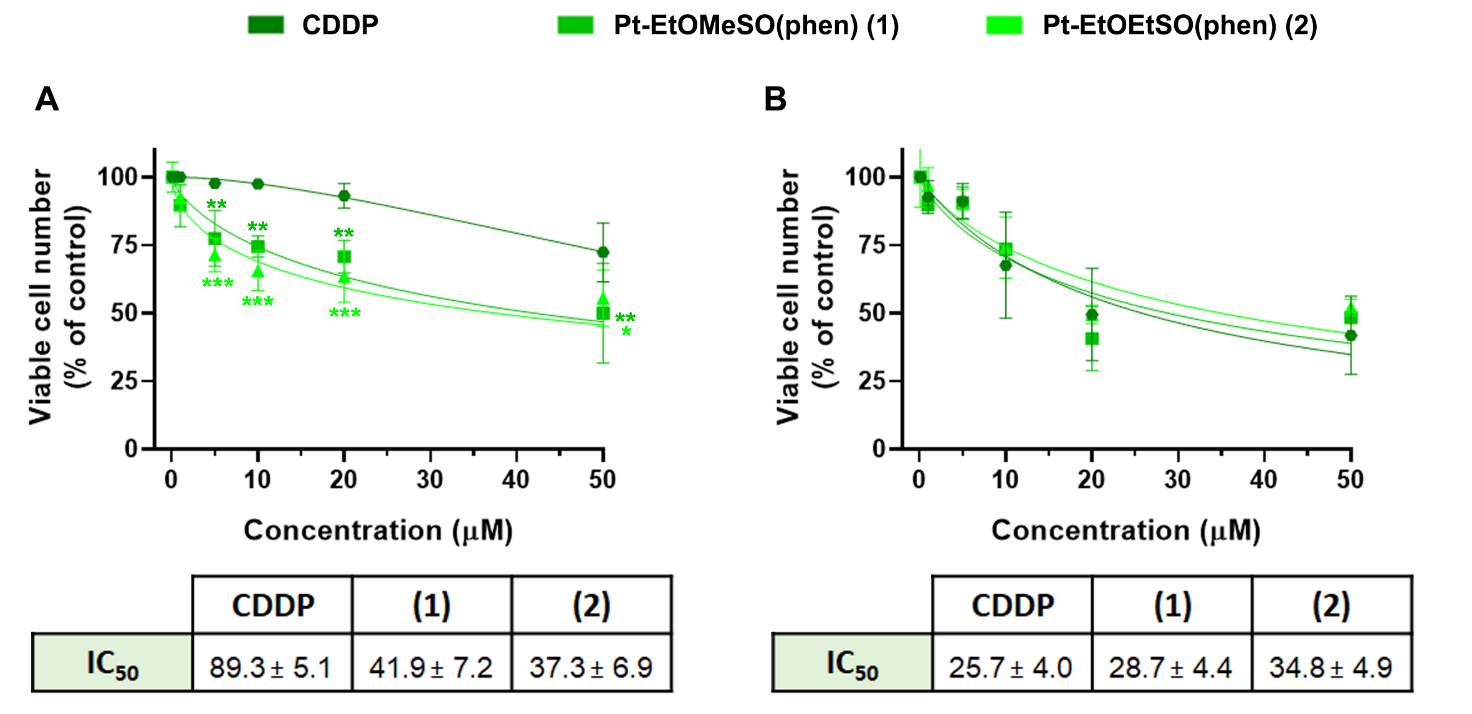


**Figure S2. Cytotoxic effects of *cis*-[PtCl_2_(NH_3_)_2_] (in short CDDP), [Pt(η^1^-C_2_H_4_OR)(DMSO)(phen)]^+^ (in short Pt-EtORSOphen; R = Me, 1; Et, 2), on normal cells.** Normal renal cells (HK-2) were exposed to varying concentrations (1 to 50 μM) of CDDP, **1**, and **2** for (A) 24 and (B) 48 h. The IC_50_ values were calculated and are presented in the tables as means ± standard deviation from eight replicate wells per microliter plate, repeated three times. Asterisks indicate values that are significantly different from CDDP for the same concentration and treatment time (*p < 0.05; **p < 0.01; ***p < 0.001).

**
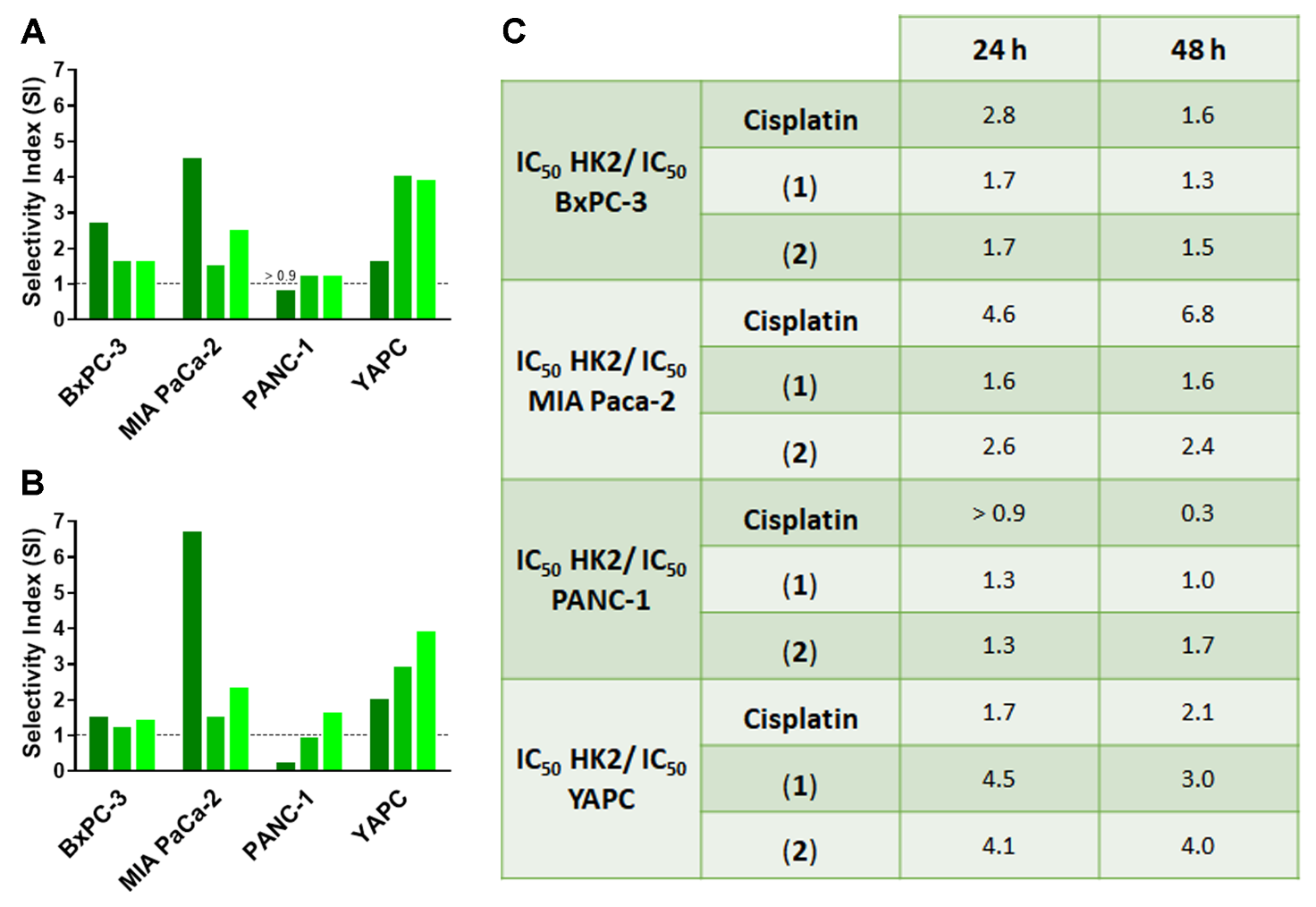
**

**Figure S3. The selectivity index (SI) values of CDDP, Pt-EtOMeSOphen (1), and Pt-EtOEtSOphen (2).** PDAC cells were exposed to different concentrations of Pt(II) complexes for 24 and 48 h. SI values were calculated using the formula: SI = (IC_50_ for normal cell line HK-2)/(IC_50_ for PDAC cancer cell lines, BxPC-3, MIA PaCa-2, PANC-1 and YAPC). An SI > 1.0 indicates a drug with higher efficacy against tumor cells compared to its toxicity against normal cells.

ù
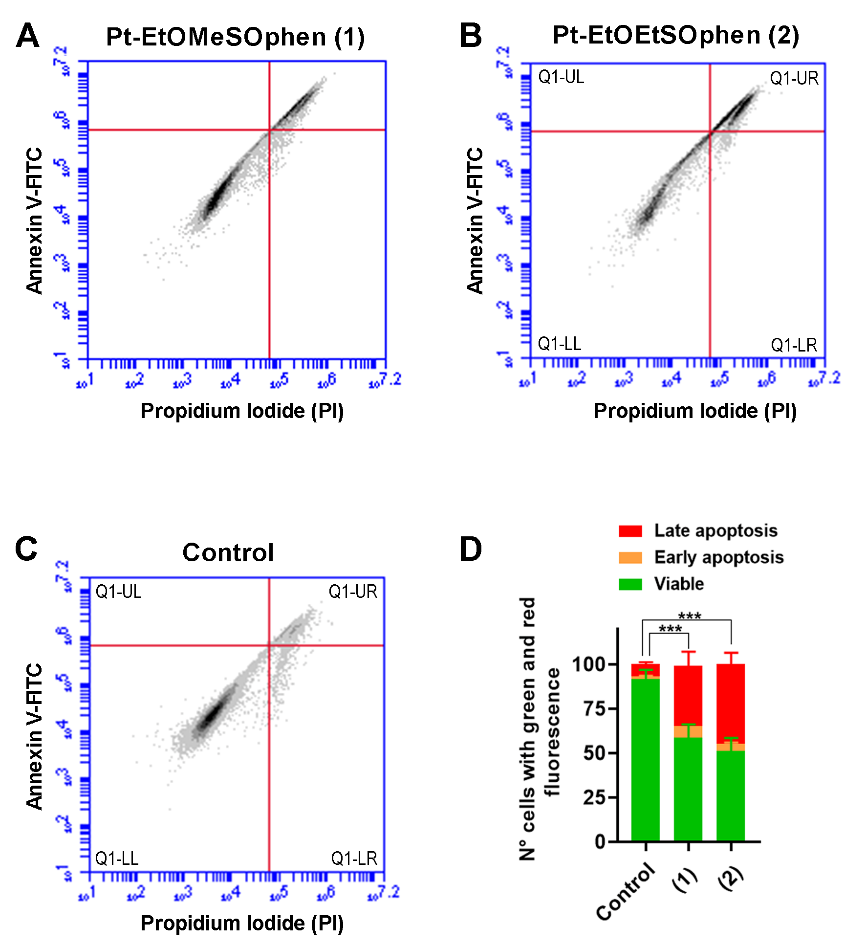


**Figure S4. Induction of apoptosis in BxPC-3 cells by Pt-EtOMeSO(phen) (1) and Pt-EtOEtSO(phen) (2).** (**A**-**C**) Cell death was measured using flow cytometry (BD Accuri C6 fow cytometer) after staining with Annexin V- FITC/PI. BxPC-3 cells were treated or not with 30 µM (**A**) complex **1** and (**B**) complex **2** for 18 h. Q1-UL, PI+ (cells undergoing necrosis); Q1-UR, annexin V-FITC + PI+ (cells in late-stage apoptosis and undergoing secondary necrosis); Q1-LR, annexin V-FITC + PI− (cells in early-stage apoptosis); Q1-LL, annexin V-FITC− PI− (living cells). The percentage of viable and dead cells was determined using BD Accuri C6 software and presented as a bar graph. (**D**) Asterisks (*** p < 0.001) indicate significantly different values of viable cells compared to the control after treatment with phen-containing complexes.


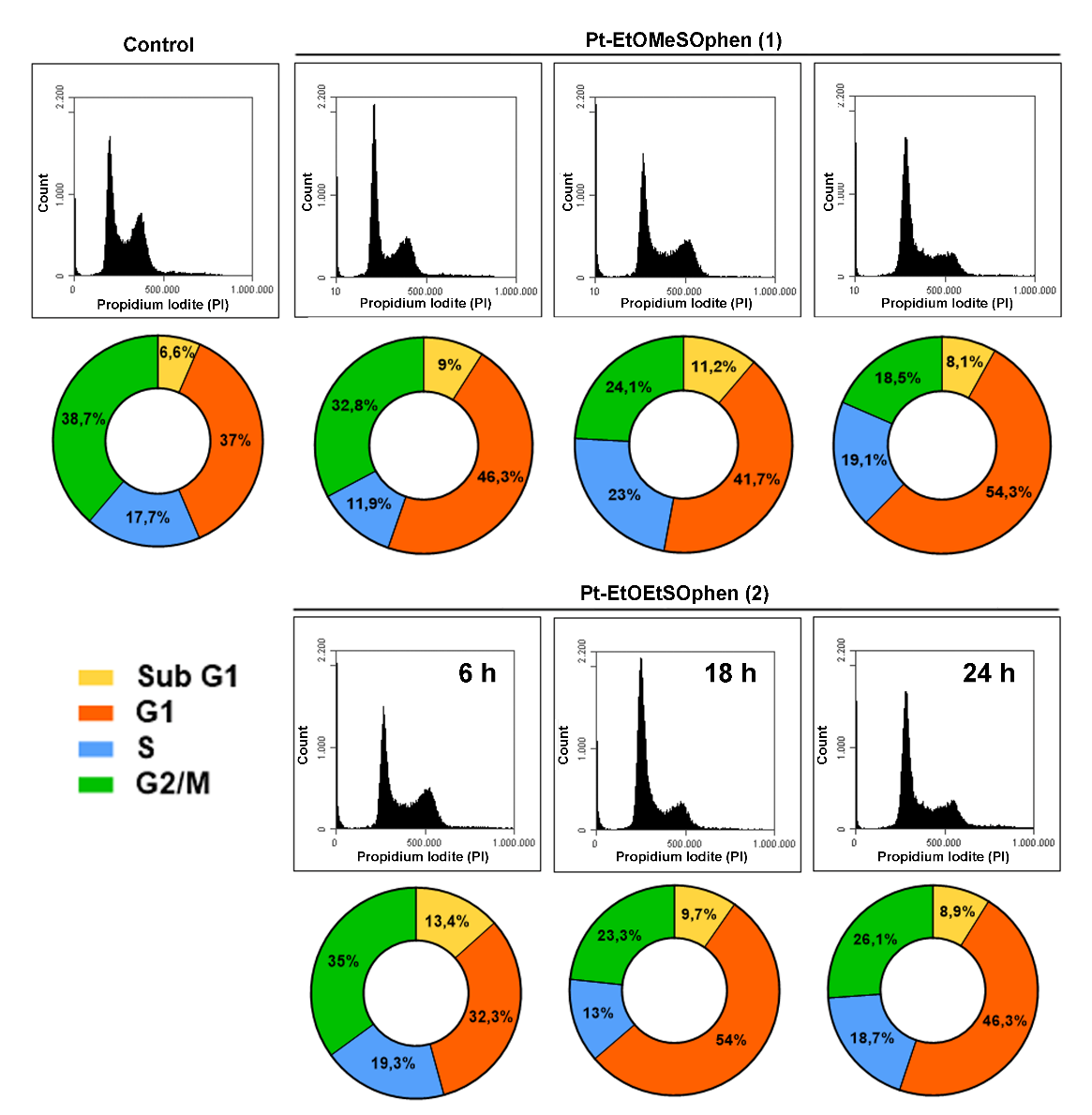


**Figure S5. Effects of Pt-EtOMeSOphen (1) and** **Pt-EtOEtSOphen (2) on cell cycle progression**. Cell cycle distribution was analyzed using flow cytometry (BD Accuri C6 flow cytometer) in PI-stained BxPC-3 cells after treatment with or without **1** and **2** for 6, 18 and 24 h. The pie charts indicate the percentages of cells in the G1, S, or G2/M phases of the cell cycle. Representative images from three independent experiments are shown.


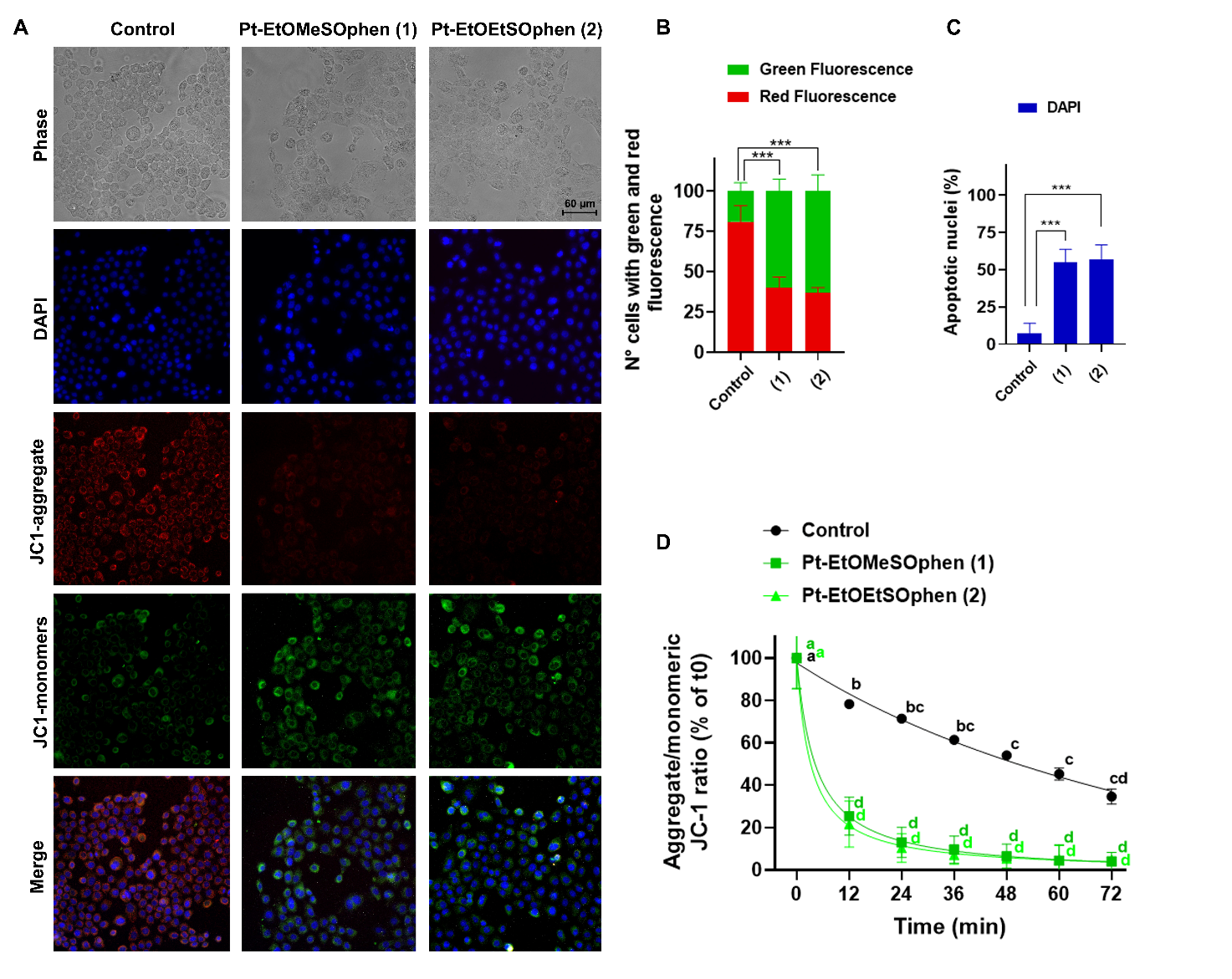


**Figure S6. Analysis of Pt(II) complex‐induced apoptosis using** **fluorescence microscopy.** (**A**-**D**) Double staining of nuclei (DAPI) and mitochondria (JC-1) was performed on BxPC-3 cells using fluorescence microscopy. Cells were treated with or without 30 μM of complexes **1** and **2** for 18 h and then stained with DAPI and JC-1 dyes. Quantification of (**A**,**B**) green/red fluorescence for measuring mitochondrial membrane potential (ΔΨ_M_) and (**A**,**C**) blue fluorescence for apoptotic nuclei was conducted using Image J software. Asterisks indicate values that are significantly different (***p < 0.001). (**D**) Measurements of J-aggregate and J-monomer fluorescence were also obtained using spectrophotometer. The data is expressed as the change in 590/520 nm fluorescence ratio induced by the treatment relative to the initial (control) 590/520 nm ratio. Results are presented as the mean ± SD of three independent experiments. Values with the same letters are not significantly different according to Tukey’s multiple comparisons test.
